# Broad-range lytic bacteriophages that kill *Staphylococcus aureus* local field strains

**DOI:** 10.1371/journal.pone.0181671

**Published:** 2017-07-25

**Authors:** Virginia Abatángelo, Natalia Peressutti Bacci, Carina A. Boncompain, Ariel A. Amadio, Soledad Carrasco, Cristian A. Suárez, Héctor R. Morbidoni

**Affiliations:** 1 Laboratorio de Microbiología Molecular, Facultad de Ciencias Médicas, Universidad Nacional de Rosario, Rosario, Santa Fe, Argentina; 2 EEA Rafaela, Instituto Nacional de Tecnología Agropecuaria (INTA), Rafaela, Santa Fe, Argentina; 3 Bioinformatics Program, Universidad Nacional de Rosario, Rosario, Santa Fe, Argentina; Cairo University, EGYPT

## Abstract

*Staphylococcus aureus* is a very successful opportunistic pathogen capable of causing a variety of diseases ranging from mild skin infections to life-threatening sepsis, meningitis and pneumonia. Its ability to display numerous virulence mechanisms matches its skill to display resistance to several antibiotics, including β-lactams, underscoring the fact that new anti-*S*. *aureus* drugs are urgently required. In this scenario, the utilization of lytic bacteriophages that kill bacteria in a genus -or even species- specific way, has become an attractive field of study. In this report, we describe the isolation, characterization and sequencing of phages capable of killing *S*. *aureus* including methicillin resistant (MRSA) and multi-drug resistant *S*. *aureus* local strains from environmental, animal and human origin. Genome sequencing and bio-informatics analysis showed the absence of genes encoding virulence factors, toxins or antibiotic resistance determinants. Of note, there was a high similarity between our set of phages to others described in the literature such as phage K. Considering that reported phages were obtained in different continents, it seems plausible that there is a commonality of genetic features that are needed for optimum, broad host range anti-staphylococcal activity of these related phages. Importantly, the high activity and broad host range of one of our phages underscores its promising value to control the presence of *S*. *aureus* in fomites, industry and hospital environments and eventually on animal and human skin. The development of a cocktail of the reported lytic phages active against *S*. *aureus*–currently under way- is thus, a sensible strategy against this pathogen.

## Introduction

The number of infections caused by multi-drug resistant pathogen bacteria has been on the rise over the last two decades; genetic evolution of enzymes, changes on bacterial cell structure and efflux pumps are some of the major mechanisms of bacterial resistance, which may also be recruited and gathered into mobile genetic elements capable of spreading more than one mechanism of resistance at the same time [1]. Besides those factors, pharmaceutical companies are limited by the low number of suitable bacterial targets amenable for drug development and by the costs imposed for such development [2,3]. However, a major effort has to be made to produce new ways to control bacterial pathogens both at a prophylactic level and for infection treatment. One of those pathogens, *Staphylococcus aureus* is a member of the human microbiota that can potentially cause a large number of infectious processes ranging from bothersome skin infections (such as folliculitis, boils and impetigo) to serious deep infections in bones, joints, heart, lungs and central nervous system, being frequent cause of osteomyelitis, septic arthritis, endocarditis, pneumonia and meningitis as well as toxin related diseases [4–9]. Over the last decades, multi-drug resistant *Staphylococcus aureus* strains were frequently identified as the origin of hospital and community infections [10,11]. Although drugs such as trimethoprim-sulfamethoxazole and vancomycin are still largely active on circulating *Staphylococcus aureus* strains, resistance to vancomycin has already been reported, causing concerns of the possible reduction or loss of activity of this antibiotic in the close future [12]. At this point, the use of bacteriophages or some of their enzymes encoded in their genomes as specific anti-bacterials is a revitalizing option [13,14]. Bacteriophages (phages in short) are viruses that prey on and kill bacteria, having a remarkable specificity for genus and even species. Phages usually fall in two categories: a) lytic, in which case the infecting phage replicates inside the bacteria and lyses the cell releasing its progeny, and b) temperate, in which case the phage replicates along with the bacteria, either integrated in the chromosome or as an extra-chromosomal plasmid-like genetic element. Lytic phages are a very attractive, eco-friendly biological anti-bacterial weapon. Due to the importance of *S*. *aureus* infections worldwide, phages active on this microbe have attracted a large deal of attention, thus, there are several reports on such phages, including genomic analysis, host-range features and anti- *S*. *aureus* activity [15–18].

In this study we describe for the first time in South America a large screening for phages active on *S*. *aureus*. Among several phages isolated, we herein report the genome sequencing and annotation of a set of lytic staphylophages. Bioinformatics analysis showed that the phages are closely related to the very-well studied phage K [19–21]; displaying a broad host range against our local *S*. *aureus* strains and therefore warranting their possible use for biomedical applications.

## Material and methods

### Culture media and bacterial growth

Chapman agar was used to isolate *S*. *aureus* from clinical samples, antibiotic susceptibility tests were done on Mueller Hinton medium. Luria Bertani (LB) agar was used for strain and phage propagation medium.

### Bacterial strains

*S*. *aureus* RN4220 (*r*^*-*^*m*^*-*^, prophage free, the kind gift of R. Novick, NYU) [22] and *S*. *aureus* ATCC 25923 [23] were used as indicator strains; 44 clinical isolates from bovine or human origin (from two different states in the country) were presumptively identified as *S*. *aureus* by conventional biochemical tests (gram stain and colony morphology compatible with *S*. *aureus*). All strains were tested for coagulase and catalase production as well as by PCR amplification of *S*. *aureus* 16sRNA and *nuc* genes [24,25]. Phenotypic antibiotic susceptibility was determined by Kirby-Bauer diffusion test, except for vancomycin in which case pre-diffusion method was used [26]. PCR amplification of *mec* gene confirmed those isolates to be characterized as MRSA [24].

All the strains from bovine or human used throughout this study received from clinical facilities were anonymized by the providers; the strains isolated in our laboratory from clinical samples, described in details elsewhere [25], were handled according to the guidelines of the Ethics Committee of the School of Medicine, Universidad Nacional de Rosario.

### Bacteriophage isolation and propagation

Samples from soil and sewage treatment plants were diluted in phage buffer (2 mM CaCl_2_, 10 mM MgSO_4_, 50 mM Tris-HCl pH 7.6, 150 mM NaCl), stirred overnight at 4°C and centrifuged 5 min at 6500 rpm after which the supernatant was filtered by 0.2 μm sterilizing filters. Five hundred μL of each sample was mixed to 200 μL of a stationary phase culture of *S*. *aureus* RN4220 or *S*. *aureus* ATCC 25923 (used as indicators strains), after 10 min at room temperature, 3.3 mL of LB top agar (2 mM CaCl_2_, 10 mM MgSO_4_ and 0.4% (w/v) agar) was added to a final volume of 4 mL, gently mixed and poured on top of fresh LB plates (2 mM CaCl_2_, 10 mM MgSO_4_ and 1.2% (w/v) agar). After hardening plates were incubated 24 h at 30 ºC. The appearance of lysis plaques indicative of the presence of phages was scored by eye. Single plaques from each plate were picked by sterile toothpicks, purified and amplified using *S*. *aureus* RN4220. Titer of each lysate was determined by ten-fold dilution of the phage suspension in phage buffer, 5 μL of each dilution were spotted on a plate containing an aliquot of 100 μL (10^6^ CFU) of a fresh overnight culture of *S*. *aureus* RN4220 in 4 mL of top agar. Plates were afterwards incubated at 30ºC for 24 h before lysis plaques were counted. High titer lysate plates were done by addition of 10^6^ PFU of the desired phage to 100 μL of a fresh overnight culture of *S*. *aureus*. The mixture was gently mixed and left at room temperature for 10 min, after which top agar (3.5 mL, 2 mM CaCl_2_, 10 mM MgSO_4_ and 0.4% (w/v) agar) was added. After hardening, plates were incubated 16–18 h at 30°C. Phages were eluted from plates showing nearly confluent lysis by addition of 4 mL of phage buffer/plate, and left standing at room temperature for 12 h. The eluate was collected, centrifuged 10 min at 8500 rpm and filtered by 0.2 μm acetate cellulose filters. Usually lysates with titers of 10^11^−10^12^ PFU/mL were obtained and kept at 4°C.

### Bacteriophage characterization

Phage morphology and virion size were characterized by Transmission Electron Microscopy using a JEOL JSM 100 CXII electron microscope. Grids were negatively stained with uranyl acetate (2% w/v). Images were acquired with a Gatan Erlangshen CCD camera.

### Genome sequencing

Genomic sequence was done at a commercial local facility (INDEAR, Instituto de Agro-Biotecnología de Rosario, Rosario, Argentina) using Illumina HiSeq 1500 technology. Libraries were generated by using the Nextera® XT DNA Sample Preparation Guide Illumina (October 2012, Illumina Inc, San Diego, CA, USA). Template bacteriophage DNA was obtained by treating high-titer bacteriophage lysates (obtained with 0.4% (w/v) agarose top medium) with DNase I (1 μg/mL) and RNase (1 μg/mL) for 1 h at 37°C followed by filtration through 0.2 μm cellulose acetate filters. After this step, guanidine thiocyanate (Sigma, final concentration 800 mg/mL) was added to the cleared lysates and the mixture was gently shaked at room temperature for 2 h for full solubilization of this salt. Bacteriophage DNA was extracted from this suspension by using DNA Wizard^®^ DNA Clean-Up System (Promega) according to the manufacturer´s instructions. The bacteriophage DNA concentration was quantitated by measuring the absorbance at 260 nm and DNA integrity was checked by agarose gel electrophoresis. DNA was finally stored at -20°C until further use. The sequences were deposited in the GenBank database under accession numbers KY794641, KY794642 and KY794643.

### Bioinformatics analysis

The A5 pipeline [27] was used for assembly of genomes sequences, resulting in an average of 999-fold coverage. The hypothetical open reading frames (ORF) present in the phage genomes were predicted by using GeneMark program [28], DNAMaster (http://phagesdb.org/DNAMaster/) and RAST [29] and were manually curated. Protein similarity was evaluated with BLASTP. Structural predictions and motif searches were done with Pfam and InterProScan [30,31]. ARNold [32] was used to detect potential rho-independent terminators. Putative tRNAs were predicted using tRNA Scan-SE [33] and ARAGORN [34]. Sequences alignment were studied using ClustalW [35] and BLASTN, allowed for the pairwise comparison of phage genome sequences. Protein alignment were performed by Clustal Omega [36] and visualized using Jalview 2.10.1 [37]. ACT (Artemis Comparison Tool) [38] was used for BLASTN alignment visualization.

The comparative analyses of whole genomes of our phages with other *Myoviridae* phages (infecting *S*. *aureus*) genomes available at NCBI database (KP687431.1, JX080302.2, JX080301.2, EU418428.2, JX080303.2, NC_007066.1, NC_019448.1, KP687432.1, KR902361.1, KR908644.1, FR852584.1, NC_019726.1, NC_005880.2, NC_025416.1, JX080304.2, JX080305.2, NC_025426.1, NC_028962.1, NC_028765.1, NC_023573.1, NC_022920.1, NC_022918.1, JX875065.1, NC_023009.1, JX080300.2, KP881332.1, NC_025417.1, NC_007021.1, NC_022090.1, NC_020877.1) were performed by using Gegenees [39]. The phylogenetic network was constructed with SplitsTree 4.14.3 software [40], using NeighbornNet method. Before the global alignments could be performed, the genomes were manually colinearized, placing the arbitrary starting point at the start of the open reading frame (ORF) of the large terminase subunit gene. The genome organization of the phages and comparative BLASTN with figures were generated by CGView Comparison Tools using build_blast_atlas.sh script [41]. An alignment plot was generated by using NUCmer with default parameters [42].

The identification and analysis of putative promoters on each of our three staphylophages was done by using a Perl script (https://www.biostars.org/p/121644/) to extract intergenic sequences from all Genbank files and those in our phages. All sequences smaller than 40 bp were discarded from analysis. Afterwards the intergenic regions were analyzed for motif discovery by using MEME Suite (http://meme-suite.org/tools/meme) [43], the numbers of motifs finding with the program was set in three and the wide of motif in six. The position in the bacteriophage genomes of the sequences that matched the motifs generated by MEME were determined by MAST. Sequences were considered as putative promoters when they were positioned on the intergenic region or no more than 30 bp inside of an upstream ORF.

### Bacteriophage host range determination

The lytic activity of the bacteriophages isolated during this study was assayed on 44 local *S*. *aureus* strains of veterinary (n = 11) and human (n = 33) origin as well as control *S*. *aureus* strains from culture collections (ATCC 29740, RN4220 and ATCC 25923) [22,23,44] as described in Table 1 Indicator plates were made adding 200 μL of late log phase cultures of the strains under assay to 3.5 mL of molten top agar (0.4% w/v) in LB, mixing gently and pouring the mix on top of LBA plates. Each phage (10^4^ PFU in 5 μL aliquots) were spotted on each plate accounting for a M.O.I of 0.01; bacteriophage K (the kind gift of Dr. A. Coffey, Department of Biological Sciences, Cork Institute of Technology, Bishoptown, Cork, Ireland) was used as control. Plate reading was done by naked eye after 24–28 h at 30°C.

**Table 1:** *Staphylococcus aureus* strains and their antibiotics resistance profile.

Origin	Strains	MET	ERI	CLI	GEN	CIP
Reference strains	ATCC29740	n. d.	n. d.	n. d.	n. d.	n. d.
	RN4220	n. d.	n. d.	n. d.	n. d.	n. d.
	ATCC25923	S	S	S	S	S
Animal strains	V329	n. d.	n. d.	n. d.	n. d.	n. d.
	I1-I3-I5-I7-I8-I9-I10-I11-I13-I23	S	S	S	S	S
Human carriers (hands)	LP274	S	R	R	S	S
	LP275-LP280-LP308-LP320-LP321	S	S	S	S	S
	LP277-LP279	S	R	S	S	S
	LP281	S	S	S	R	S
Human carriers (nostrils)	C6-C32-C352	S	S	S	S	S
	C10	R	S	S	R	S
	C18	R	R	R	R	R
	C77	R	R	R	S	R
	C115-B377	R	S	S	S	S
	C136	S	R	R	S	R
	C161-C310-B399	S	R	R	S	S
	B422	R	R	R	R	S
	B426	S	S	S	R	S
Clinical samples	H1-H42-H45	S	S	S	S	S
	H2-H5-H6	R	S	S	S	S
	H10	R	S	S	R	S
	H43-H44	S	R	R	S	S
	H50	R	R	R	S	R

R and S correspond to resistance and susceptibility to the antibiotics listed, respectively. MET, methicillin ERY, erythromycin; CLI, clindamycin; CIP, ciprofloxacin; RIF, rifampicin; GEN, gentamicin. All strains assayed were susceptible to tobramycin, mupirocin and sulfomethoxazol-trimethroprim. n. d. = not determined.

## Results

### Isolation and characterization of lytic staphylophages

As part of a broad program to isolate phages active on *S*. *aureus* (thus dubbed staphylophages for short), we implemented two strategies: first, to look for lytic phages in environmental samples and second, to obtain temperate phages by inducing phage excision from lysogenic *S*. *aureus* strains from environmental, veterinary and human sources through U.V. or mitomycin treatment. Both strategies yielded several lytic and temperate phages, the latter group will be reported elsewhere. Lytic staphylophages were isolated from samples collected from sewage treatment plants and soil samples in Santa Fe and Buenos Aires, Argentina, following standard procedures as described in Materials and Methods and using *S*. *aureus* RN4220 (a prophage free strain) and *S*. *aureus* ATCC 25923 as propagating strains. In this way, eight phages were purified from different samples, two (named S24 and CG) from soil samples and the remaining six phages (named Clo2, Clo5, Clo6, Clo7, Clo9 and Clo11) detected in a sewage sample. Observation of the plaque morphology generated by these eight phages showed that Clo and CG phages yielded large (2–3 mm) very clear plaques while S24 gave smaller and turbid plaques in the indicator strains.

**Fig 1:**
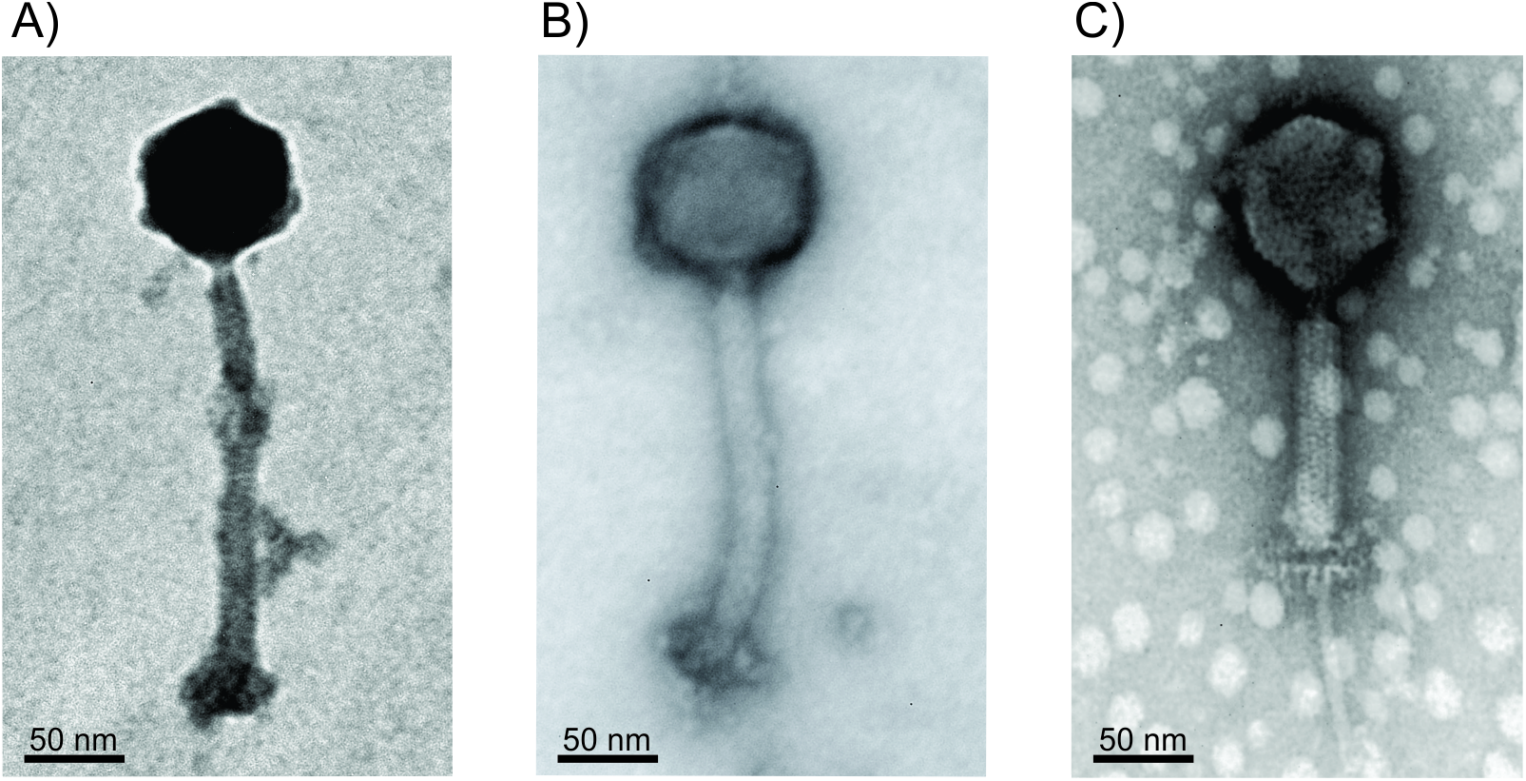
Transmission electron micrographs. Images correspond to phages, A) vB_Sau_S24, B) vB_Sau_CG and C) vB_Sau_Clo6.

The transmission electron microscopy (TEM) analysis results showed that all the staphylophages under study belong to the *Myoviridae* family, having icosahedral heads with sizes ranging from 65 to 90 nm and long contractile tails from 200 to 230 nm in the extended state (Fig 1). Some of the electron micrographs showed phages with contracted tails displaying a double baseplate, as has been reported for other *Myoviridae* phages [45].

After this analysis, we followed the nomenclature guidelines proposed by Kroprinski *et al*. [46,47], thus our phages were renamed vB_Sau_Clo2, vB_Sau_Clo5, vB_Sau_Clo6, vB_Sau_Clo7, vB_Sau_Clo9, vB_Sau_Clo11, vB_Sau_S24 and vB_Sau_CG, nomenclature that will be used throughout the rest of this manuscript when necessary.

### Genome analysis

#### General features

The complete genomes of all isolated staphylophages were sequenced at a commercial local facility. The size of the double stranded DNA genomes containing terminally redundant ends was determined after assembling the raw DNA sequencing data;ranging from 139,997 bp (vB_Sau_S24) to 143,734 bp (for all vB_Sau_Clo phages), having an almost identical G+C% content, coding capacity (CDS) and gene density (Table 2). A more detailed comparison of the vB_Sau_Clo phages revealed that not only they share the same exact genome length but also the G+C% content (30.86%) and tRNA number (n = 1). Upon most detailed analysis using BLASTN and Artemis Comparison Tool (ACT) we confirmed that all the vB_Sau_Clo phages were in fact identical and thus we kept one (vB_Sau_Clo6) as representative for further genetic and biological characterization. The two remaining phages, vB_Sau_S24 and vB_Sau_CG showed slightly different features from the ones displayed by vB_Sau_Clo6; moreover, when compared using Artemis to phage K—a very well characterized lytic staphylophage- all our phages are very similar in general genomic characteristics as shown in Table 2.

**Table 2:** General genomic characteristics of phages vB_Sau_CG, vB_Sau_S24, vB_Sau_Clo6 and phage K.

Bacteriophage	Genomic size (bp)	G+C percent	CDS	Coding percentage	Genic density (gen/Kbp)	tRNA	Average length (bp)
vB_Sau_CG	142934	30.51	224	90.4	1.57	5	577
vB_Sau_S24	139997	30.86	209	89.8	1.49	2	602
vB_Sau_Clo6	143734	30.86	213	90.5	1.48	1	610
Phage K	148317	30.39	233	88.9	1.57	4	566

The annotation of the phages genomes was performed by using DNAMaster and RAST programs. The overall genome organization of the isolated staphylophages yielded an organization comparable to other staphylophages, with four major modules encompassing genes corresponding to cell lysis, phage morphogenesis, DNA packaging and DNA replication and transcription. This analysis located 214 coding sequences in vB_Sau_Clo6, 211 in vB_Sau_S24 and 229 in the vB_Sau_CG phage genome. The analysis of presence of tRNAs using ARAGORN and tRNA-Scan showed that vB_Sau_CG encoded five (tRNA-Asp, tRNA-Phe, tRNA-Trp, tRNA-Met and tRNA-His), vB_Sau_S24 two (tRNA-Asp and tRNA-Arg), and vB_Sau_Clo6 only one (tRNA-Asp) (Table 2 and S1 Table).

The direct strand encoded for 160, 158 and 167 ORF and the reverse strand encoded for 54, 53 and 62 ORF, for vB_Sau_Clo6, vB_Sau_S24 and vB_Sau_CG, respectively. Comparison to sequences available in public databases showed that vB_Sau_Clo6 had 13/146 (9.8%) hypothetical proteins with no homology to any phage protein in the GenBank database; two of these proteins had homologues in our phages (vB_Sau_S24 and vB_Sau_CG). Phage vB_Sau_S24 had 10/140 (7.1%) hypothetical proteins with no homologues in public databases, of those nine had high homology to hypothetical proteins encoded by vB_Sau_Clo6 and one was similar to a protein present in vB_Sau_CG. Moreover, vB_Sau_CG had only 4/161 ORFs (2.5%) encoding hypothetical proteins with no homology to phages on databases, although one of these, ORF157, was homologous to one in our phages vB_Sau_S24 and vB_Sau_Clo6. Coding density was comparable for these phages, with 9.5–9.7% of non-coding sequences dispersed in their genomes. A viral strategy to pack information in a reduced amount of genomic room is to have overlapping genes, this trait was verified in our three phages where we could identify 23, 26 and 30 ORFs (for vB_Sau_S24, vB_Sau_Clo6 and vB_Sau_CG, respectively) overlapping the 3´-end of the upstream ORF and the 5´-end of the downstream ORF. The genomes of the three phages that we analyzed did not contain genes encoding virulence-associated or toxic proteins such as enterotoxin A, leukocidin and exfoliative toxin [48,49], a desirable trait if potential use in biotechnology applications is envisioned.

#### Promoter and terminator analysis

**Fig 2:**
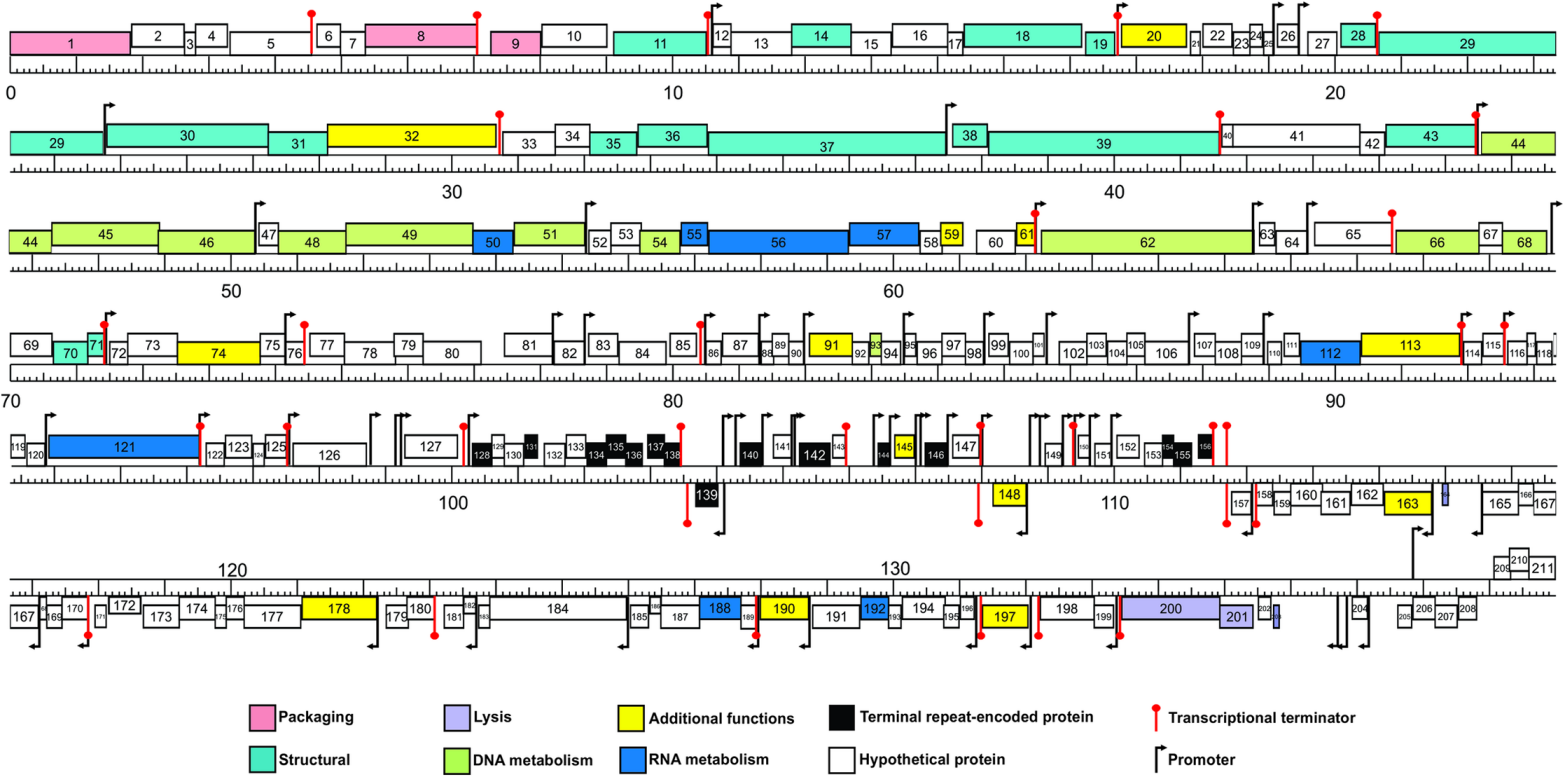
Genome organization of the phage vB_Sau_S24. ORFs functions are shown in different colors. The 72 putative promoters were represented by an arrow and 34 putative rho-independent transcriptional terminators by stem loops.

**Fig 3:**
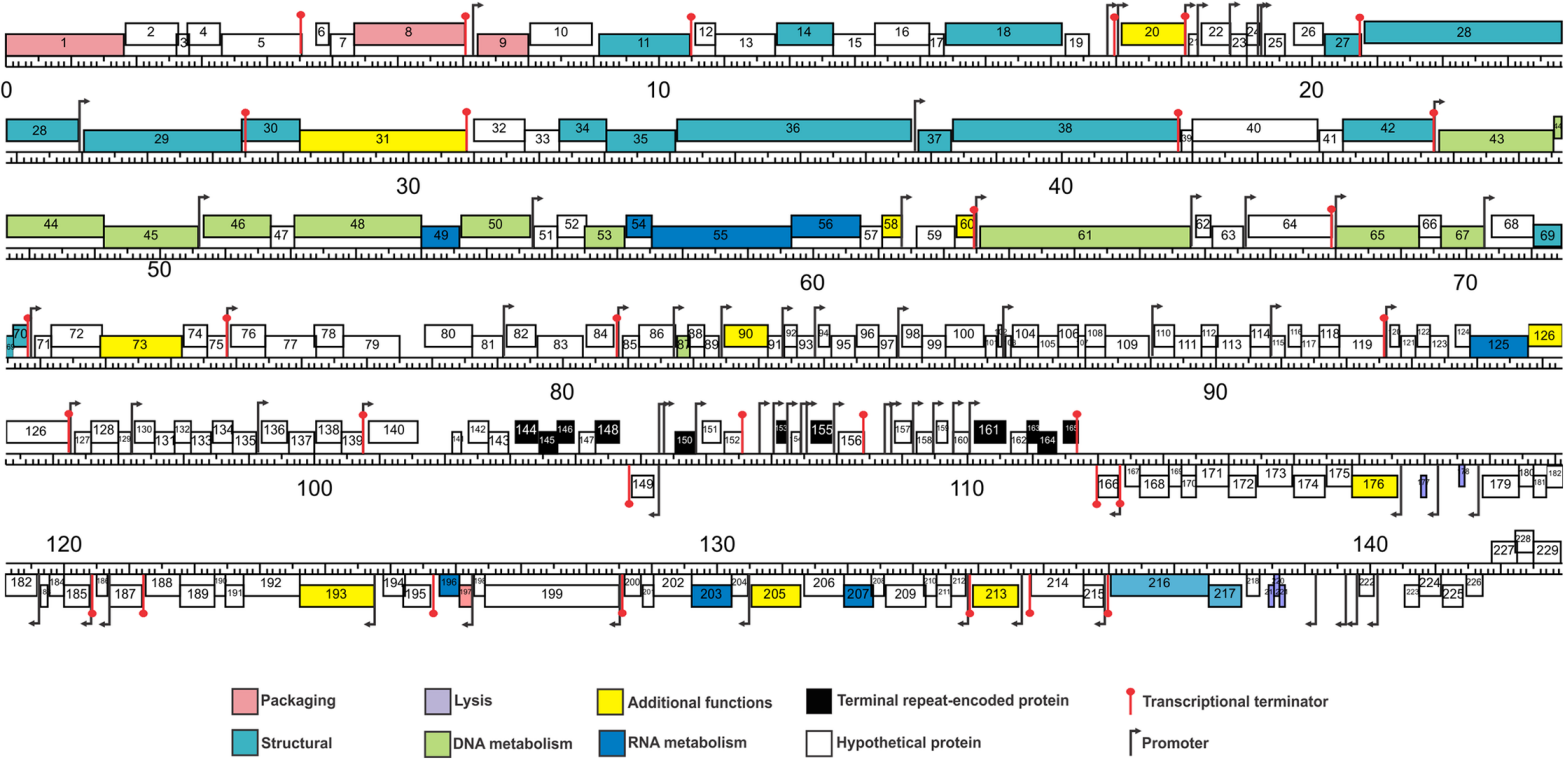
Genome organization of the phage vB_Sau_CG. ORFs functions are shown in different colors. The 70 putative promoters were represented by an arrow and 31 putative rho-independent transcriptional terminators by stem loops.

**Fig 4:**
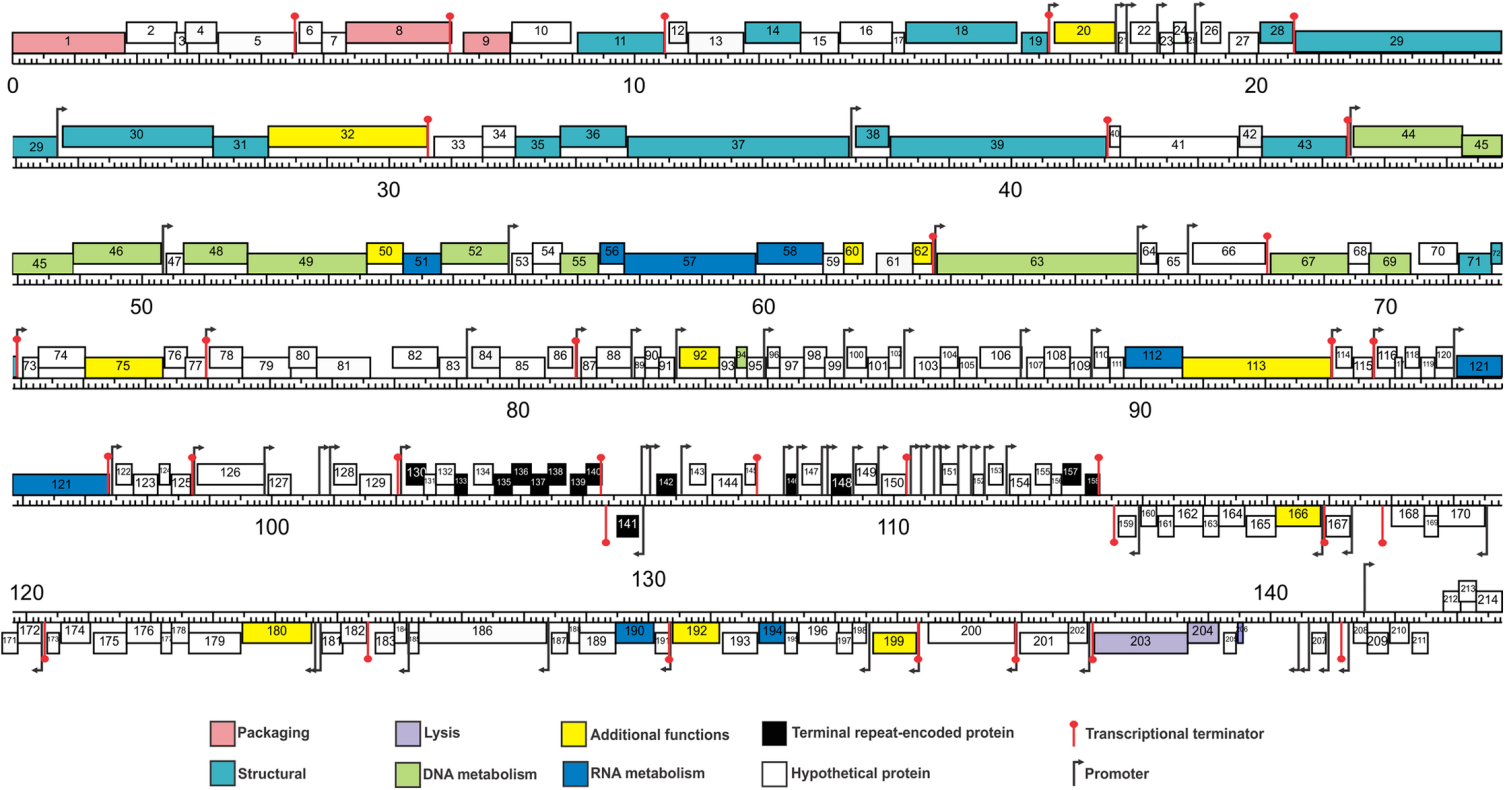
Genome organization of the phage vB_Sau_Clo6. ORFs functions are shown in different colors. The 70 putative promoters were represented by an arrow and 31 putative rho-independent transcriptional terminators by stem loops.

After genome comparison, we searched for regulatory consensus sequences in the intergenic regions of the three phages using MEME suite [43]. Thus, we generated a different consensus motif for each phage that was in agreement with the consensus sequences for *S*. *aureus* σ^70^ dependent promoters (S1 Fig) and were very similar to the consensus sequence for the putative promoters of the ISP phage [50]. These consensus sequences had a conserved -35 box whereas the -10 box was more variable; the spacer regions had a length of 17 nucleotides. The screening of promoters along the phages genomes was performed using MAST and visual inspection. A total of 72 putative promoters were identified in vB_Sau_S24 and 70 in each vB_Sau_CG and vB_Sau_Clo6 (Figs 2–4). The distribution of those promoters in each strand and their position was comparable for the three phages under study. The search of putative rho-independent transcription terminators pinpointed 31 putative terminators for the vB_Sau Clo6 and vB_Sau_CG phages, and 34 for the vB_Sau_S24 phage (Figs 2–4).

Phages not encoding their own RNA polymerase must use the host´s for the transcription of all their early genes and subsequently could modify it by using alternative σ factors to direct the transcription of middle and late genes [21]. Scrutiny of the genome sequences also pinpointed genes encoding putative alternative σfactors, a feature that is also displayed by other staphylophages such as A5W (*sig*), K (ORF94), G1 (ORF56), ISP (ORF69) and Sb-1 (ORF140). The search for homologous genes in our phages, led to the identification of two putative genes coding for alternative σ factors, ORF68 and ORF69 for phages vB_Sau_S24 and vB_Sau_Clo6 respectively, and ORF67 for phage vB_Sau_CG; all of them were closely related. Another bacteriophage strategy to subdue and use the host´s cell machinery is the take-over of the transcriptional factory; in this aspect, it has been demonstrated that phages use anti-sigma factors (*i*.*e*. gpORF67, phage G1) to interact with the primary σ factor (σ^70^), therefore preventing the transcription of bacterial promoters and early phage promoters and allowing for transcription of late genes [51]. During our analysis we were able to identify such type of putative anti-σ factors encoded in genes ORF50 for vB_Sau_S24, ORF51 for vB_Sau_Clo6 and ORF49 for vB_Sau_CG.

#### Comparative genomics

**Fig 5:**
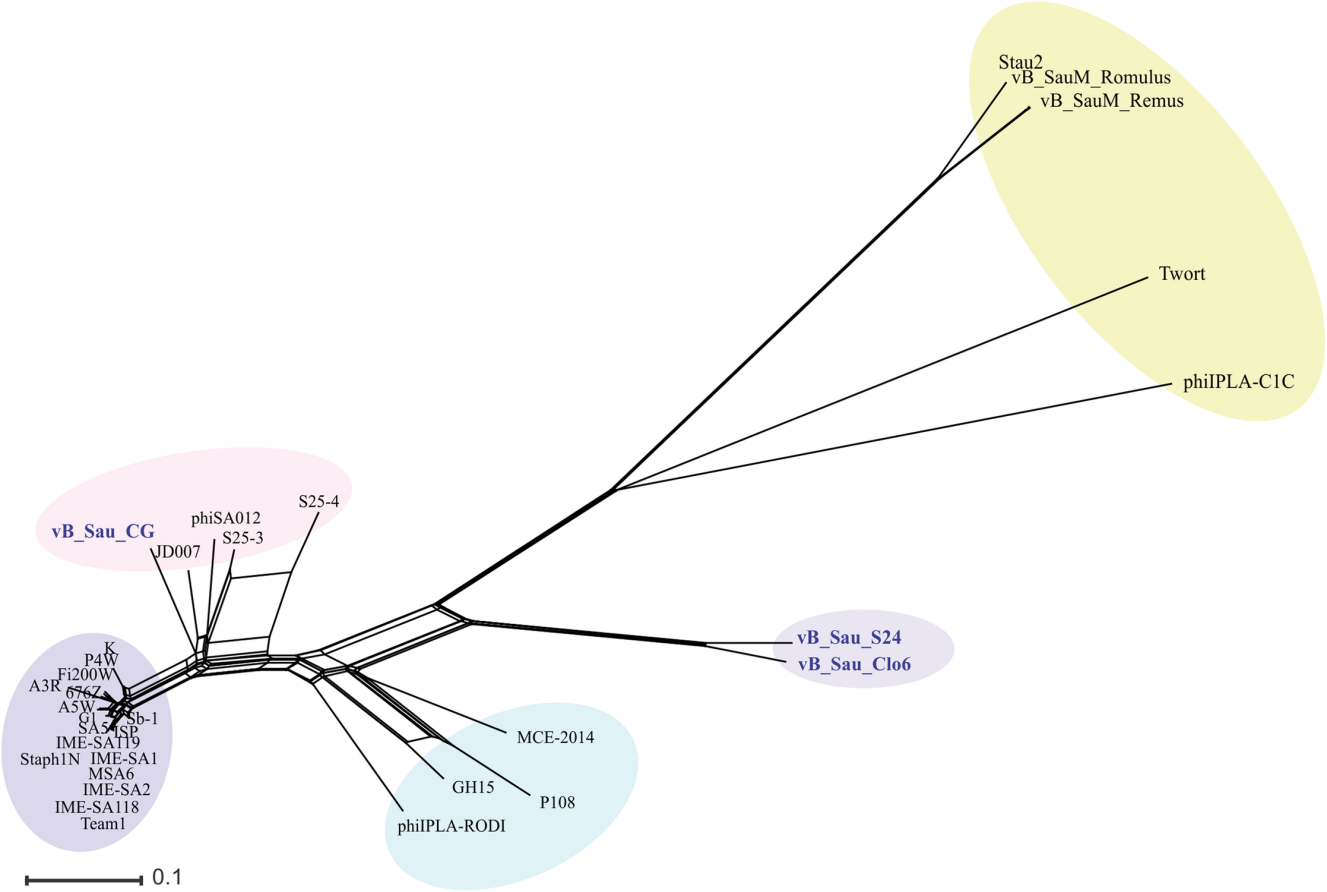
SplitsTree networks analysis of the *Myoviridae* family bacteriophages. Thirty genomes available in database were aligned by Gegenees and the network was built with SplitsTree4 by the Neighbor-Net method. Five phylogenetic groups (clusters) were identified. vB_Sau_S24 and vB_Sau_Clo6 clustered together forming a separate group.

**Fig 6:**
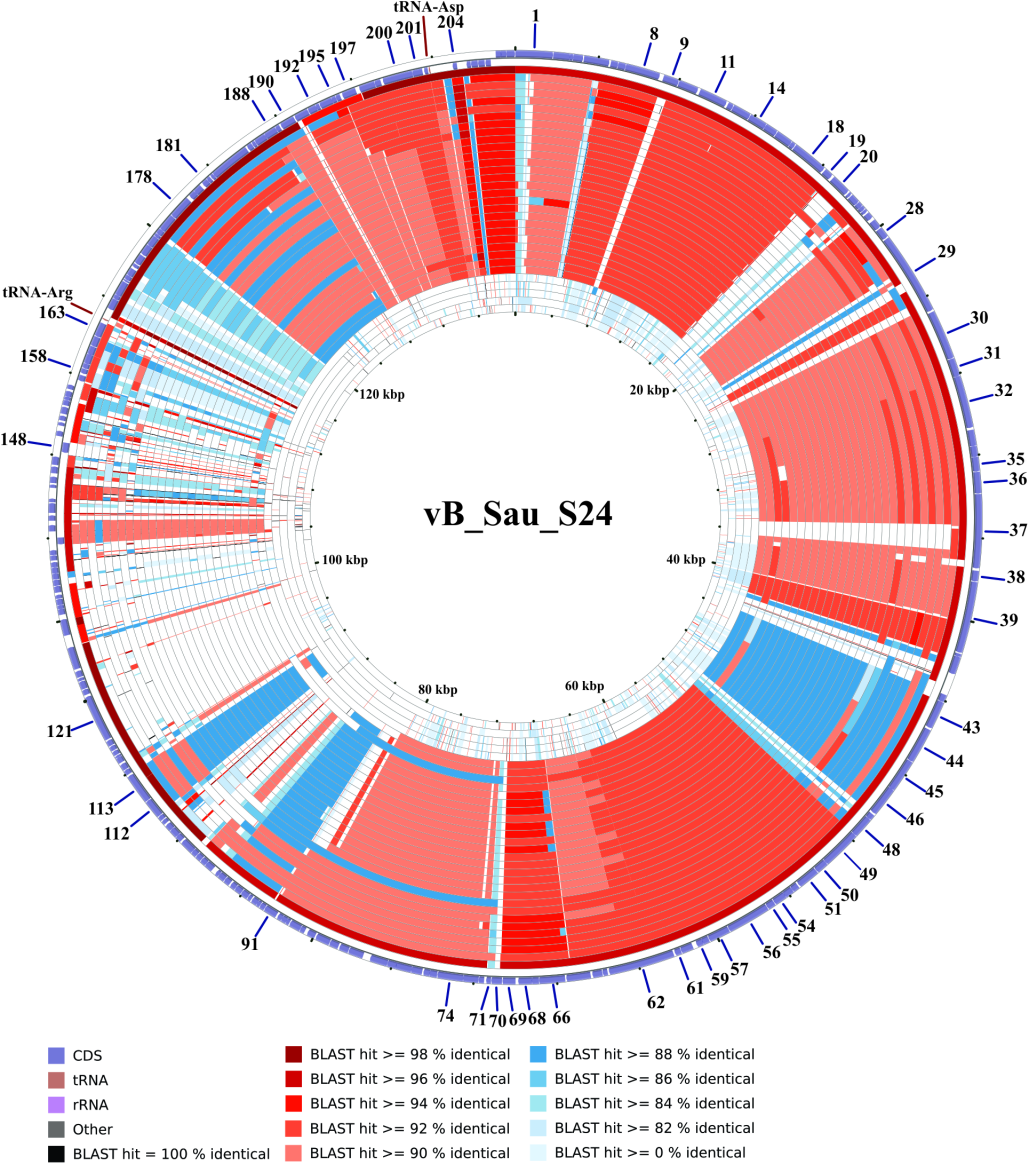
CCT map comparing the genome of phage vB_Sau_S24 to other *S*. *aureus* myobacteriophages. The most external ring in the graph corresponds to vB_Sau_S24 used as a reference genome. The next 32 rings correspond to BLASTN alignment of the each genome analyzed, the color correspond to the percent of sequence similarity (see below panel).

**Fig 7:**
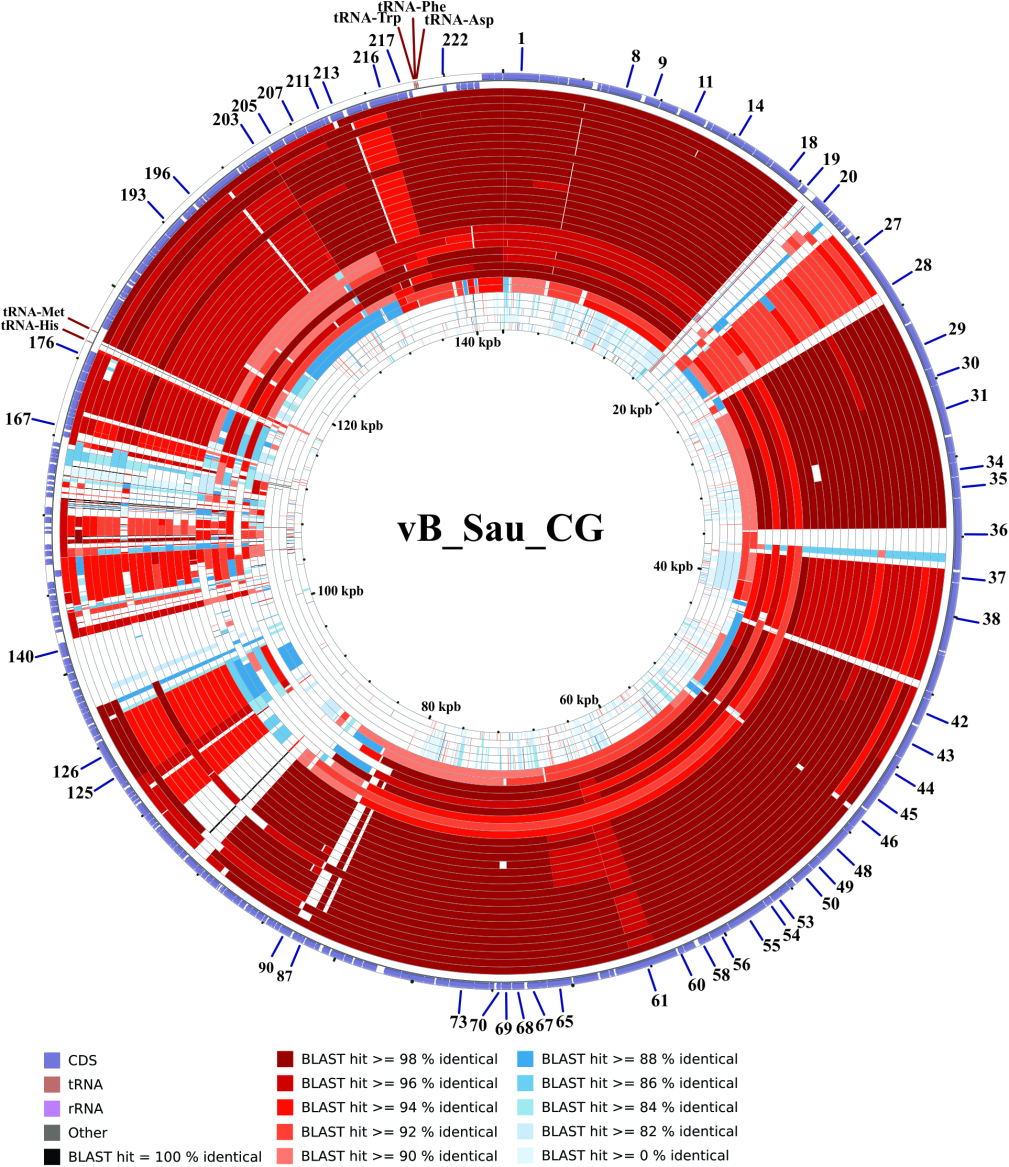
CCT map comparing the genome of phages vB_Sau_CG to other *S*. *aureus* myobacteriophages. The most external ring in the graph corresponds to vB_Sau_CG used as a reference genome. The next 32 rings correspond to BLASTN alignment of the each genome analyzed, the color correspond to the percent of sequence similarity (see below panel).

**Fig 8:**
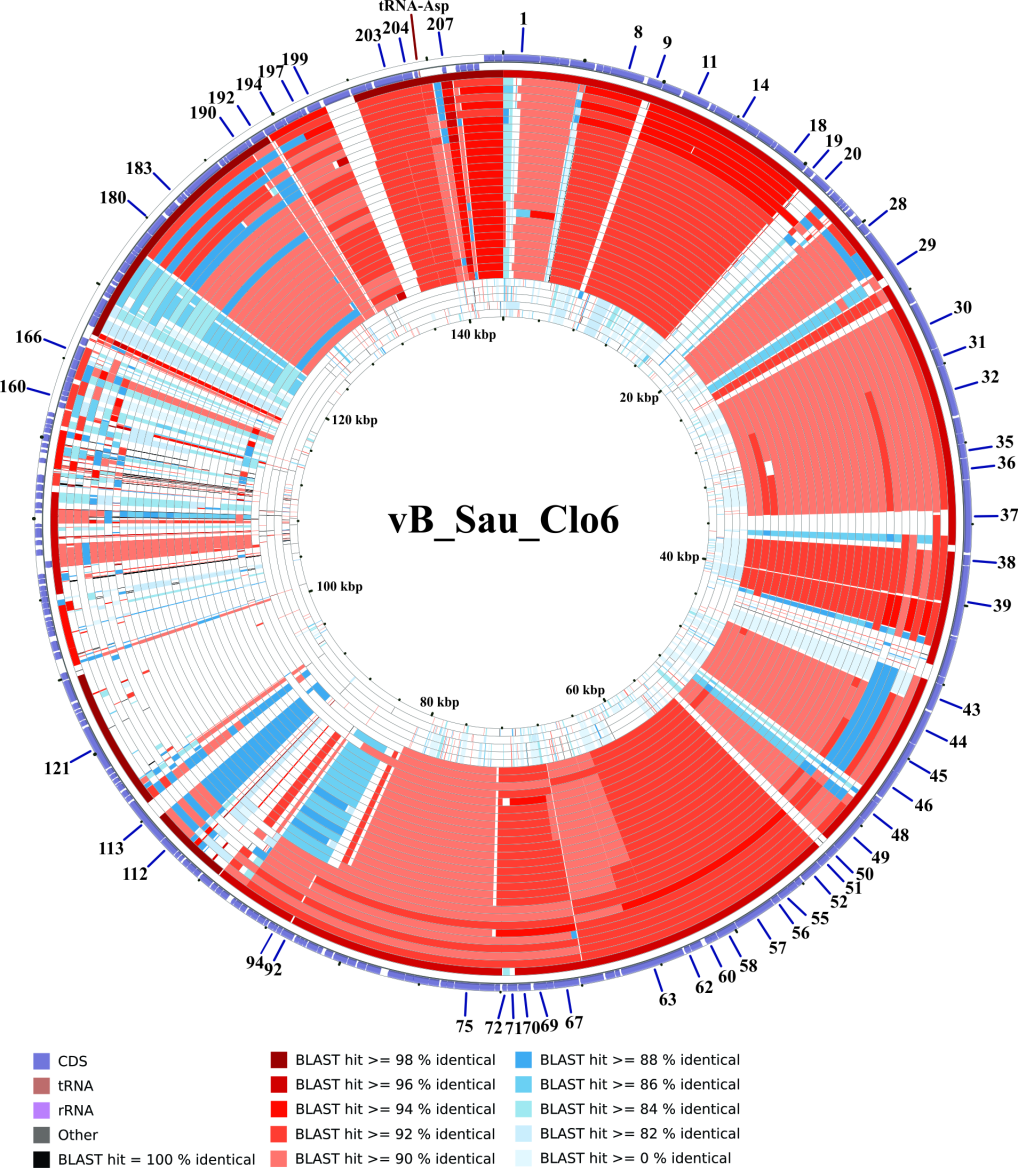
CCT map comparing the genome of phage vB_Sau_Clo6 to other *S*. *aureus* myobacteriophages. The most external ring in the graph corresponds to vB_Sau_Clo6 used as a reference genome. The next 32 rings correspond to BLASTN alignment of the each genome analyzed, the color correspond to the percent of sequence similarity (see below panel).

The phylogenetic relationship of our phages to other phages belonging to the *Myoviridae* family was performed using Gegenees software [39] and Splitstree4 [40]. As shown in Fig 5, the phages could be divided in five groups (clusters), with vB_Sau_S24 and vB_Sau_Clo6 forming themselves a separate group and vB_Sau_CG belonging into a different phylogenetic group. Notwithstanding that, the identity percent analysis using ClustalW when aligning our phages to phage K (taken as reference since it is one of the best characterized members of this group) showed a high level of identity, 82%, 91% and 81% for phages vB_Sau_S24, vB_Sau_CG and vB_Sau_Clo6, respectively. A pairwise comparison of the genomic sequence of our three phages with that of phage K was carried out using Nucmer from MUMmer 3.23 package [42,52]. The results, shown as a dot plot alignment, revealed extensive homology (S2 Fig) with differences in the Long Terminal Region (LTR). Comparison of genetic sequences through CGView Comparison Tool [41] using BLASTN, showed differences such as the absence of specific genes containing introns and homing endonucleases, present in phage K and several other staphylococcal *Myoviruses* (Figs 6–8) [53]. Also this analysis showed that our phages shared zones with high similarity to the majority of phages aligned (dark zones) and less similar in the LTR region, in agreement with NUCmer analysis (S2 Fig). The LTR borders are defined by the *treA* and *bofL* ORFs in each phage. Moreover, the vB_Sau_CG phage is the most similar one to the other phages analyzed (Fig 7) in agreement with the phylogenetic analysis performed by Gegenees (Fig 5).

The phage morphogenesis module encompassing genes related to the synthesis of components of the virion structure is generally described as separated into five submodules; packaging, head morphogenesis, head-tail connection, tail morphogenesis and lysis. As usually found in staphylococcal Twort-like phages, genes forming each different functional submodule are not physically together, i.e., genes belonging to the tail morphogenesis submodule are placed throughout the genome and separated by proteins of unknown function [54]; our phages contained 16 proteins in this module. The tape measure (TmpC), that determines the tail length of the phages (ORF29 of staphylophages vB_Sau_Clo6 and vB_Sau_S24 and ORF 28 of vB_Sau_CG) encoded proteins of 1341, 1353 and 1376 aminoacids, respectively. A search for conserved domains in TmpC was performed using InterproScan, leading to the identification of a putative lysozyme-like domain in phages vB_Sau_S24 (from AA 1150 to 1245) and vB_Sau_Clo6 (from AA 1139 to 1233). This domain was previously described in bacteriophage vB_SauS-phiIPLA35 [55], it was demonstrated that the domain encoded a functional muramidase that may be involved in bacterial cell wall degradation during infection [56].

The cell lysis module comprises the holin and endolysin genes whose products are required for proper release of mature virions from infected cells [57]. Bioinformatics analysis of the endolysins encoded by our phages using Pfam, revealed the presence of two enzymatically active domains (EADs) at the N-terminus, a CHAP domain having endopeptidase activity (PF05257) and in a center of the protein an amidase domain AMI2 (PF01510) (the most frequently described in *Staphylococcus* endolysins) that is linked to a C-terminal cell wall binding domain (CBD) belonging to the SH3-5 type [58]. Interestingly, the endolysin encoding genes of our three phages have no introns, opposite to what has been described for other phages like K, G1, ISP and A5W among others [59].

#### Presence of hypothetical introns and inteins

The presence of Group I introns interrupting protein encoding genes has been demonstrated in the genome of several phages infecting *Staphylococcus* and belonging to the *Myoviridae* family [59]. In fact, several well studied phages such as Romulus and Remus, K and G1 contain introns in genes encoding helicase, ribonucleotide reductase large subunit, endolysin and DNA polymerase. Another gene reported as containing introns is *tmpA*, encoding a tail tube subunit; in phage Twort this gene is interrupted by three introns [60]. In spite of this, we could not detect introns in those genes in our phages. However, the tail morphogenetic protein *tmpF* (ORF37 in vB_Sau_S24 and vB_Sau_Clo6 and ORF36 in vB_Sau_CG), displayed a higher size than what was described in other staphylophages; when compared to reported *tmp*F sequences from other staphylophages such as phage K, we noticed an internal fragment spanning 145 AA in ORF37 and 146 AA in ORF36. A comparable insertion is also present in the ORF005 of phage Twort, which encodes a *tmp*F gene [53]. When the insertions were compared, homology was only detected in the C-end half of phage Twort ORF005 protein. This may be indicative of the presence of an intron in *tmp*F although direct confirmation by protein analysis will be required. We also found that the nicotinamide phosphoribosyl transferase of phage vB_Sau_Clo6 (ORF113) has a putative length of 796 AA, 307 AA larger than the one determined for other staphylophages, including vB_Sau_CG (ORF126) and vB_Sau_24 (ORF113). BLASTP analysis showed an insertion from amino acid 312 to 578; this feature is not shared by any staphylococcal phage in the NCBI database. Protein domain analysis of the translated full sequence of this insertion, performed with InterproScan showed a Hint domain (Hegdehog/intein) at coordinates 310 to 399; this domain is involved in protein splicing, and was also reported for the helicase gene in phage Twort [60]. The alignment of the insertion that contains the Hint domain using BLASTP showed high homology with ribonucleotide-diphosphate reductase alpha subunit of phages vB_SauM_Romulus [61] and Stau2 [62].

Endonucleases encoding genes have been detected in phage vB_Sau_CG, in which ORF140 encodes for one of such enzymes, belonging to the GIG-YIG superfamily and displaying an identity of 44% to one ORF such present in the genome of *E*. *faecium*. In the same line, ORF20 contains a putative intron-encoded nuclease displaying 73% of similarity to a protein present in *S*. *aureus* phage 812. The corresponding ORF20 in phages vB_Sau_Clo6 and vB_Sau_S24 also contains an intron-encoded nuclease with 99% of identity to the one present in the staphylococcal phage ΦSA012 (Accession Nº NC_023573).). Another putative endonuclease was identified by Pfam analysis of the ORF50 of phage vB_Sau_Clo6 this ORF possesses an HNH endonuclease domain from AA69 to 111 (IPR003615). BLASTP alignment showed that the encoded protein of ORF50 share good homology (87% of identity) with a hypothetical protein from the staphylococcal phage pSco-10 (Accession Nº ANH50485.1).

In summary, the phages we have characterized display similarity to Twort related phages but also have distinctive features when it comes to their content of possible introns and endonucleases. Analysis of the proteins of these phages will be required in order to ascertain whether those insertions encode for true introns and functional endonucleases.

### Determination of host range activity for staphylophages

The use of alternative ways to control bacterial pathogens is of obvious convenience both in industrial settings as well as in human and animal health care due to the decreasing efficacy of currently used anti-bacterial drugs and the potential effects exerted by residues of disinfectants. One of our major aims through this work was to assemble a mixture of genetically characterized phages with broad killing activity on local *S*. *aureus* strains. To that end we assayed our phages activity against 44 *S*. *aureus* isolated strains and 3 reference strains, of which 11 were MRSA (Table 1). Both vB_Sau_Clo6 and vB_Sau_CG phages were highly active on the strains under assay, with an 89% and 81% of killing activity, respectively (Table 3). Interestingly the activity of our phages was comparable to that of phage K, a well-known, broad range lytic staphylophage which in our hands displayed activity on a 79% of the total strains assayed. Finally, vB_Sau_S24 was the less efficacious phage, displaying activity on only 15% of the strains tested.

**Table 3:** Host range analysis. The *S*. *aureus* strains were separated by origin. The orange cells indicate the strains lysis by the corresponding phage. The lysis percentages of each phage are mentioned in the last column.

*S*. *aureus* strains and origin		Bacteriophages			
		vB_Sau_S24	vB_Sau_CG	vB_Sau_Clo6	Phage K
**Reference strains**	**ATCC29740**				
	**RN4220**				
	**ATCC25923**				
**Animal strains**	**V329/I11**				
	**I1**				
	**I3**				
	**I5**				
	**I7**				
	**I8**				
	**I9/I23**				
	**I10**				
	**I13**				
**Human carriers (hands)**	**LP320/280/277/321**				
	**LP281**				
	**LP308**				
	**LP274**				
	**LP275**				
	**LP279**				
**Human carriers (nostrils)**	**C6/C32**				
	**C136**				
	**C161**				
	**C310**				
	**B377/C352**				
	**C10/C18**				
	**C77**				
	**C115**				
	**B422**				
	**B426**				
	**B399**				
**Clinical samples**	**H42**				
	**H1**				
	**H44/H5/H50**				
	**H43**				
	**H45/H2**				
	**H6**				
	**H10**				
**Percentage (%)**		**15**	**81**	**89**	**79**

In this regard, phage host range is determined by the interaction of receptor proteins located on the bacterial cell surface and **R**eceptor **B**inding **P**roteins (RBP) present in the tail fibers of the phage. Habann *et al*, showed that RBPs of phage A511 (active on various members of the genus *Listeria*) and staphylococcal phages ISP and Twort (Gp108, Gp40 and Gp17 respectively) are located on the short fibers of the tail [63]. Takeuchi *et al* recently demonstrated that the phage genomic region spanning ORF103-ORF105 in staphylococcal phage ΦSA012 is conserved in Twort-like phages and are critical to determine the phage host range [64]. Based on this information we searched our phages for ORFs homologues to ΦSA012 ORF103, in this way we identified vB_Sau_Clo6 ORF41, vB_Sau_CG ORF40 and vB_Sau_S24 ORF41, all of them with an E-value = 0. Given the role of this ORF in host range, we performed an alignment of ORF40 and 41 in our phages and gp146 in phage K, finding that the N-end aminoacid sequence of the protein is more conserved between all these phages than the C-terminal region which is more variable (S3 Fig). However, in spite of the sequence differences found, none of them could explain by bioinformatics analysis alone the narrow host range exhibited by phage vB_Sau_S24. Only two of the tested field strains were resistant to all phages assayed, supporting our contention that we have isolated lytic bacteriophages capable of forming part of an anti-*Staphylococcus aureus* bacteriophage cocktail.

## Discussion

Lytic bacteriophages are catching the eye of researchers and industry as possible materials to counterattack the rise of antibiotic-resistant bacteria, among them, *S*. *aureus* certainly is one of the most dangerous and difficult to treat. Several publications describe the isolation and thorough physical and genomic characterization of bacteriophages specifically active against *S*. *aureus* strains towards the end of their biotechnological applications [65–67]. However there is a paucity of information about the bacteriophages that are present in our region as well as how refractory to already described anti-*Staphylococcus* phages our local strains would be. Thus we carried on a search for local lytic bacteriophages with activity against both human and animal *S*. *aureus* strains circulating in Argentina. As a general strategy we searched for lytic bacteriophages from environmental (sewage waters, ponds, and soil samples), and human (through nares swabs) sources. We simultaneously gathered a large number of local *S*. *aureus* strains of human, animal and environmental origin to be used as hosts to test the bacteriophages host range. Initially our search yielded six different phages isolated from sewage waters and two from soils samples, however further analysis showed that the first group were identical phages isolated by two different operators from the same large volume sample and thus we kept only one for further studies. The three phages were classified as members of the *Myoviridae* family, having genomes of 140–150 kbp determined by genomic sequencing. Thus, based on genome size and gene organization they also belong to Class III as defined by Kwan [60], a group that also contains phages Twort, K, A5W, ISP, Sb-1 and G1. The phylogenetic relationship with other bacteriophages of the *Myoviridae* family indicated that vB_Sau_S24 and vB_Sau_Clo6 grouped into a different cluster more distantly related to the rest of the family members. The general genome features (G+C content, coding capacity, gene organization) related them to phage K, one of the best characterized anti- *S*. *aureus* lytic phages described. A large number of putative promoters recognized by *S*. *aureus* σ^70^ were located on the phages genomes, as well as anti-σ factors and an alternative σ factor closely resembling those described for other *S*. *aureus Myoviridae* phages. One of the most distinctive singularities of the phages isolated in this study is the lack of introns in genes containing them in other *Staphylococcus Myoviridae* phages; in fact previous studies have illustrated that there are introns and/or inteins in class III phages [60]. An example of that is shown by lysin and polymerase genes of several Class III phages, excluding Twort [21]. In addition, several other genes of phage Twort have been reported to contain introns or inteins [53,60,68]. Interestingly a recently described phage, related to phages G1, ISP, A5W, Sb-1 and K, GH15, is lacking introns in genes encoding critical enzymatic functions [69]. All the introns and inteins present in the phages mentioned above were absent in our phages, indicating an intron loss. However, analysis of our set of phages revealed the presence of insertions only in genes with hypothetical functions, except for the insertions located in *tmp*F and a gene encoding a nicotinamide phosphoribosyl transferase. The comparative analysis of the phage genome sequences reported here not only provides convincing evidence for the diversity of staphylococcal myovirus phages but also offers new clues to intron change in phages.

In a worldwide situation where anti-bacterial drugs activity is jeopardized by the bacterial mechanisms of resistance, a biological approach based on the utilization of bacteriophages holds promise. In our area of interest, staphylophages seem to be a sensible way to attack *S*. *aureus* without displaying a broad, non-selective killing effect on the rest of the microbiota or on the microbial world linked to human activities through the environment. The current literature demonstrates the feasibility of the use of one or more staphylophages to reduce bacterial load on surfaces, fomites, and even in animal models [50,66,67]. Moreover, phages enzymes active on peptidoglycan linkages have been studied thoroughly leading to at least one commercial formulation already in the market for the treatment of MRSA infections in humans (Staphefekt, Micreos, The Netherlands, www.micreos.nl). However, due to their protein nature, the current use is as an adjuvant to treat skin conditions such as rosacea and acne [70]. In this context, we tested our three new lytic staphylophages on a large number of *S*. *aureus* strains; finding an attractive range of activity of both vB_Sau_CG and vB_Sau_Clo6 on *S*. *aureus* strains regardless of their origin and their antibiotic resistance profile. Activity was comparable to the one displayed by phage K, a very encouraging result that supports our idea of an industrial application once phage features such as stability to pH, temperature and salts is determined [21]. On the contrary, vB_Sau_24 showed a very selective activity on few strains and seems not to add value to a cocktail. Analysis of ORF41 in phages vB_Sau_Clo6 and vB_Sau_S24, ORF40 in vB_Sau_CG and gp146 in phage K revealed aminoacid differences in the variable C-end region; however we could not establish a direct link to the narrow host range of phage vB_Sau_S24, specially given its relatedness to phage vB_Sau_Clo6. Besides the fact that other ORFs also involved in the interaction with the bacterial cell receptors (such as ORFs 42 and 43 of vB_Sau_S24 and vB_Sau_Clo6) were not analyzed at this time, it is clear that experimental evidence (such as gene swapping between phages) will be needed to gain insight in the molecular features determining the behavior as a narrow or broad host range phage. Interestingly, point mutations affecting the minor tail protein encoded by ORF22 of the mycobacteriophage Halo (capable of infecting *Mycobacterium smegmatis*) modified its host range, becoming able to infect *Mycobacterium tuberculosis* [71]. This strongly suggests that a few aminoacid variations may dramatically change host range even inter species.

Of note, two *S*. *aureus* strains, both frequently isolated from cow mastitis (I11 and V329) were neither destroyed by our phages nor by phage K. Besides the identification of lytic phages with broad killing activity on local *S*. *aureus* strains, these results create a useful frame for its efficacy improvement, as we have identified two circulating strains resistant to all our staphylophages that we have tested. In this matter, more work on the isolation of phage mutants gaining the ability to destroy those strains is warranted.

Given the relatedness of the phages genome sequences and gene organization as well as their comparable and high activity on *S*. *aureus* strains both reported in this manuscript and in several others from different geographical locations, we suggest that evolutionary forces helped shape and maintain a core of features necessary for successful propagation and extended activity of lytic *Myoviridae* phages on *S*. *aureus* strains.

## Supporting information

S1 Table List of predicted proteins encoded by our three phages and staphylococcal *Myoviruses* G1, K, ISP and Twort. The (+) and (-) indicates the presence or absence of the protein, respectively. The accession numbers of phages G1, K, ISP and Twort are, NC_007066, NC_005880, FR852584, NC_007021.
(DOCX)

S1 Fig Regulatory consensus sequences by MEME. Promoter consensus sequences were generated using intergenic regions. Putative -35 and -10 regions were identified for each sequence with a spacer region of 17 nucleotides between them.
(TIF)

S2 Fig Dot-plot alignment using NUCmer. This figure showed the aligned segments with dots or lines. The nucleotide sequence of the bacteriophage K genome is represented on the X-axes and the genomes of vB_Sau_Clo6, vB_Sau_CG and vB_Sau_S24 are represented on Y-axis. Gaps are zones with no homology, which correspond to the LTR region.
(TIF)

S3 Fig Sequences comparison of the RBPs homologues in phages vB_Sau_S24, vB_Sau_Clo6, vB_Sau_CG and K. Alignment of the ORF41 of phages vB_Sau_S24 and vB_Sau_Clo6, ORF40 of vB_Sau_CG and gp146 of phage K was performed using Clustal Omega with default parameters. The results were visualized with Jalview 2.10.1 program; the color pattern shows percentage identity between proteins.
(TIF)
