# Correction: Broad-range lytic bacteriophages that kill *Staphylococcus aureus* local field strains

**DOI:** 10.1371/journal.pone.0187387

**Published:** 2017-10-27

**Authors:** Virginia Abatángelo, Natalia Peressutti Bacci, Carina A. Boncompain, Ariel F. Amadio, Soledad Carrasco, Cristian A. Suárez, Héctor R. Morbidoni

The fourth author’s middle initial is incorrect. The correct name is: Ariel F. Amadio. The correct citation is: Abatángelo V, Peressutti Bacci N, Boncompain CA, Amadio AF, Carrasco S, Suárez CA, et al. (2017) Broad-range lytic bacteriophages that kill *Staphylococcus aureus* local field strains. PLoS ONE12(7): e0181671. https://doi.org/10.1371/journal.pone.0181671
