# Supplementary material for: Broad-range lytic bacteriophages that kill Staphylococcus aureus local field strains
Source: PLoS One. 2017 Jul 25;12(7):e0181671. doi: 10.1371/journal.pone.0181671 (PMC5526547; doi:10.1371/journal.pone.0181671)
Supplement: S1 Table — The (+) and (-) indicates the presence or absence of the protein, respectively. The accession numbers of phages G1, K, ISP and Twort are, NC_007066, NC_005880, FR852584, NC_007021. (DOCX) [file pone.0181671.s001.docx]

|  | **Putative function** | **Average length (AA)** | **vB_Sau_S24** | **vB_Sau_CG** | **vB_Sau_Clo6** | **G1** | **K** | **ISP** | **Twort** |
| --- | --- | --- | --- | --- | --- | --- | --- | --- | --- |
| **Packaging** | Terminase large subunit | 605 | 1 | 1 | 1 | + | + | + | + |
|  | Portal protein | 563 | 8 | 8 | 8 | + | + | + | + |
|  | Prohead protease | 257 | 9 | 9 | 9 | + | + | + | + |
| **Structural** | Major capsid protein | 463 | 11 | 11 | 11 | + | + | + | + |
|  | Capsid protein | 158-293 | 14 | 14 | 14 | + | + | + | + |
|  | Major tail sheath protein | 587 | 18 | 18 | 18 | + | + | + | + |
|  | Tail tube protein | 142 | 19 | 19 | 19 | + | + | + | + |
|  | Tail morphogenetic protein (TmpB) | 178 | 28 | 27 | 28 | + | + | + | + |
|  | Tail tape measure | 1341-1377 | 29 | 28 | 29 | + | + | + | + |
|  | Tail murein hydrolase (TAME) | 808 | 30 | 29 | 30 | + | + | + | + |
|  | Peptidoglycan Hydrolase (TmpE) | 296 | 31 | 30 | 31 | + | + | + | + |
|  | Baseplate wedge subunit (BmpA) | 234 | 35 | 34 | 35 | + | + | + | + |
|  | Baseplate morphogenetic protein (BmpB) | 348 | 36 | 35 | 36 | + | + | + | + |
|  | Tail morphogenetic protein (TmpF) | 1019 | 37 | 36 | 37 | + | + | + | + |
|  | Baseplate morphogenetic protein (BmpC) | 173 | 38 | 37 | 38 | + | + | + | + |
|  | Adsorption-associated tail protein (TmpG) | 1152 | 39 | 38 | 39 | + | + | + | + |
|  | Tail fiber protein | 458 | 43 | 42 | 43 | + | + | + | + |
|  | Tail morphogenetic protein (TmpH) | 73-173 | 70 | 69 | 71 | + | + | + | + |
|  | Tail morphogenetic protein (TmpI) | 75 | 71 | 70 | 72 | + | + | + | + |
| **DNA manipulation** | DNA helicase A | 582 | 44 | 43 | 44 | + | + | + | + |
|  | Rep protein | 537 | 45 | 44 | 45 | + | + | + | + |
|  | DNA helicase B | 480 | 46 | 45 | 46 | + | + | + | + |
|  | Recombination exonuclease A | 345 | 48 | 46 | 48 | + | + | + | + |
|  | Recombination exonuclease B | 639 | 49 | 48 | 49 | + | + | + | + |
|  | DNA primase | 355 | 51 | 50 | 52 | + | + | + | + |
|  | Resolvase | 202 | 54 | 53 | 55 | + | + | + | + |
|  | DNA polymerase A | 1008-1072 | 62 | 61 | 63 | + | + | + | + |
|  | Repair recombinase | 418 | 66 | 65 | 67 | + | + | + | + |
|  | RNA polymerase sigma factor | 220 | 68 | 67 | 69 | + | + | + | + |
|  | DNA sliding clump inhibitor | 58 | 93 | 87 | 94 | + | - | + | - |
| **RNA manipulation** | Anti-sigma factor | 198 | 50 | 49 | 51 | + | + | + | + |
|  | Ribonucleotide reductase, stimulatory protein | 143 | 55 | 54 | 56 | + | + | + | + |
|  | Ribonucleotide reductase, large subunit | 704 | 56 | 55 | 57 | + | + | + | + |
|  | Ribonucleotide reductase, minor subunit | 349 | 57 | 56 | 58 | + | + | + | + |
|  | Ribose-phosphate pyrophosphokinase | 302 | 112 | 125 | 112 | + | + | + | - |
|  | RNA ligase | 758 | 121 | - | 121 | - | - | - | + |
|  | Nucleoside triphosphate pyrophosphohydrolase | 101 | 181 | 196 | 183 | + | + | + | - |
|  | DNA/RNA ligase | 298 | - | - | - | + | + | + | - |
|  | Nucleoside 2-deoxyribosyltransferase | 208 | 188 | 203 | 190 | + | + | + | + |
|  | Ribonuclease | 141 | 192 | 207 | 194 | + | + | + | + |
| **Lysis** | Endolysin (N-acetylmuramoyl-L-alanine amidase) | 495 | 200 | 216 | 203 | + | + | + | + |
|  | Holin | 167 | 201 | 217 | 204 | + | + | + | + |
| **Additional functions** | Putative intron-encoded nuclease | 324 | 20 | 20 | 20 | - | - | - | + |
|  | Glycerophosphoryl diester phosphodiesterase | 849 | 32 | 31 | 32 | + | + | + | + |
|  | Thioredoxin | 106 | 59 | 58 | 60 | + | + | + | + |
|  | DNA binding protein | 101 | 61 | 60 | 62 | + | + | + | + |
|  | Intron encoded protein (nuclease) I-KsaI | 166 | - | - | - | + | + | + | + |
|  | Intron encoded protein (nuclease) I-KsaII | 170 | - | - | - | + | + | + | - |
|  | Intron encoded protein (nuclease) I-KsaIII | 269 | - | - | - | + | + | + | + |
|  | Ig like protein | 210 | 69 | 68 | 70 | + | + | + | + |
|  | Metallophosphoesterase | 416 | 74 | 73 | 75 | + | + | + | + |
|  | RuBisCO small subunit | 215 | 91 | 90 | 92 | - | - | - | - |
|  | Nicotinamide phosphoribosyl transferase (NadV) | 381-489 | 113 | 126 | 113 | + | + | + | - |
|  | Group I intron endonuclease | 245 | - | 140 | - | - | - | - | - |
|  | HNH homing endonuclease | 162-194 | 148 | - | 50 | + | + | + | - |
|  | BofL | 78-82 | 158 | 167 | 160 | + | + | + | + |
|  | Serine/Threonine protein phosphatase | 235 | 163 | 176 | 166 | + | + | + | + |
|  | AAA family ATPase | 372 | 178 | 193 | 180 | + | + | + | - |
|  | PhoH related protein | 246 | 190 | 205 | 192 | + | + | + | + |
|  | Trancriptional regulator | 70-76 | 195 | 211 | 197 | + | + | + | + |
|  | Transglycosylase | 210-232 | 197 | 213 | 199 | + | + | + | + |
|  | Intergenic region ORF protein | 72 | 204 | 222 | 207 | + | + | + | + |
| **tRNAs** | tRNA-Asp |  | + | + | + | + | + | + | - |
|  | tRNA-Arg |  | + | - | - | - | - | - | - |
|  | tRNA-Phe |  | - | + | - | + | + | + | - |
|  | tRNA-Trp |  | - | + | - | + | + | + | - |
|  | tRNA-Met |  | - | + | - | + | + | + | + |
|  | tRNA-His |  | - | + | - | - | - | - | - |
