# Supplementary figures and images for: Broad-range lytic bacteriophages that kill Staphylococcus aureus local field strains
Source: PLoS One. 2017 Jul 25;12(7):e0181671. doi: 10.1371/journal.pone.0181671 (PMC5526547; doi:10.1371/journal.pone.0181671)

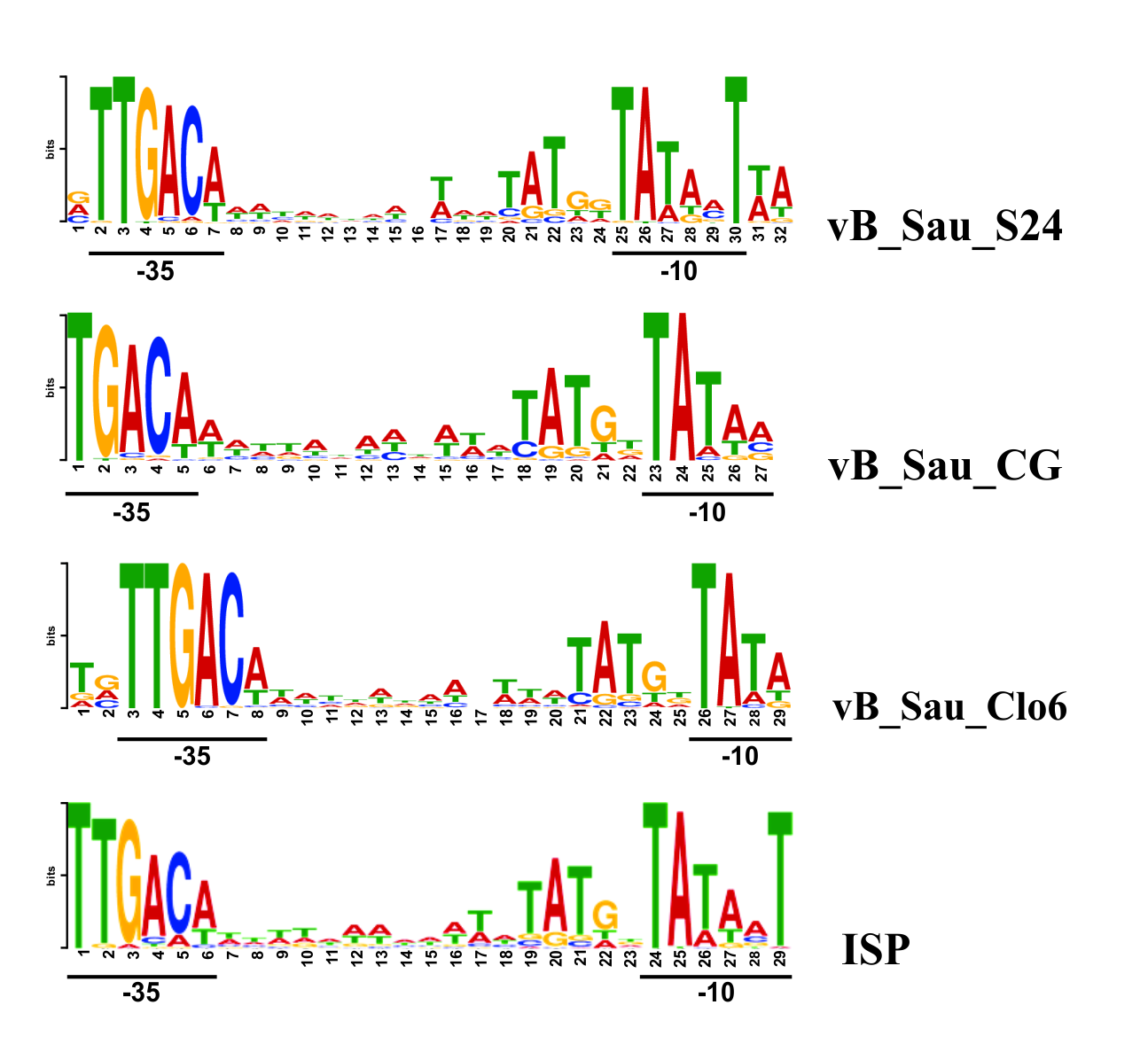

Supplement: S1 Fig — Promoter consensus sequences were generated using intergenic regions. Putative -35 and -10 regions were identified for each sequence with a spacer region of 17 nucleotides between them. (TIF) [file pone.0181671.s002.tif]

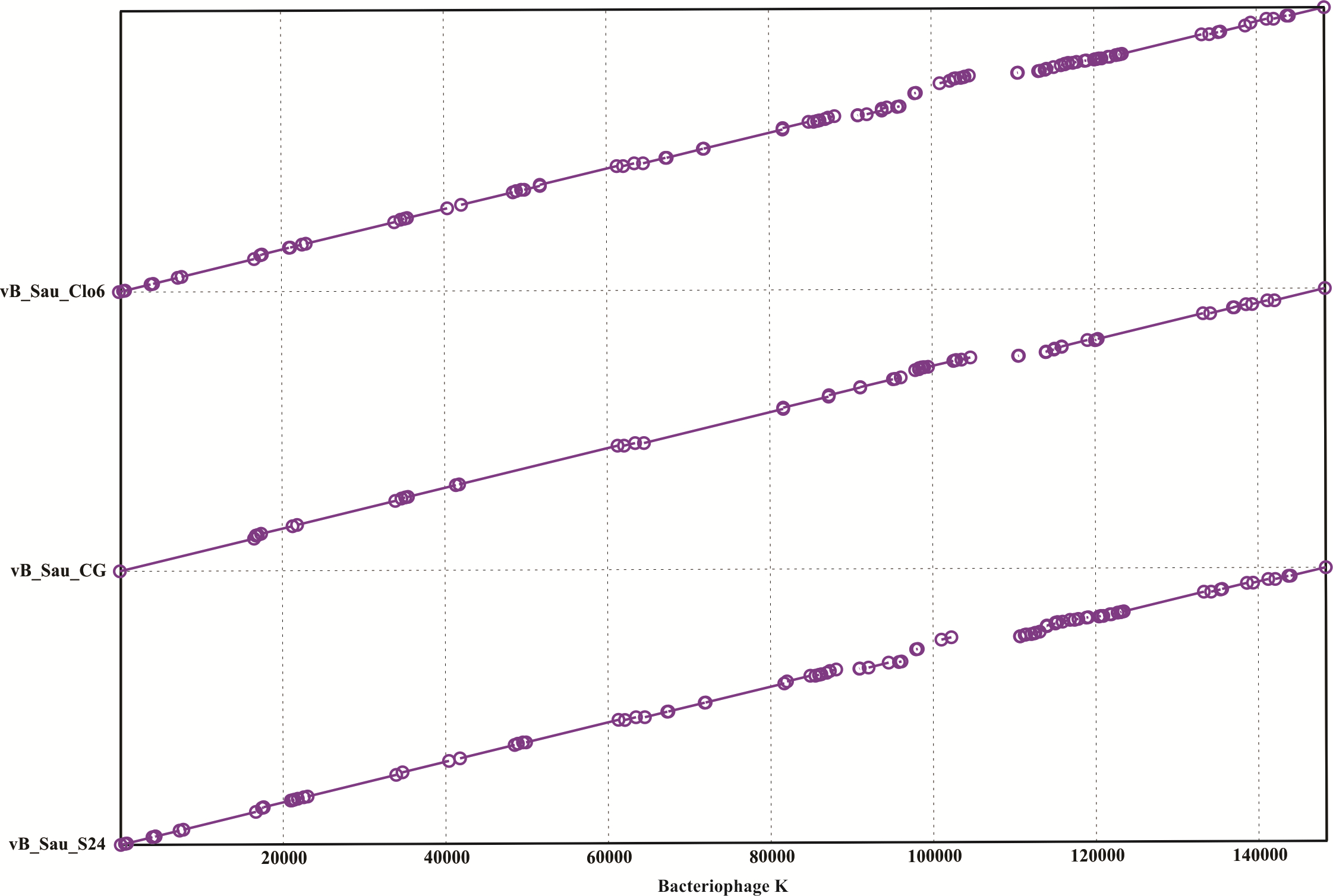

Supplement: S2 Fig — This figure showed the aligned segments with dots or lines. The nucleotide sequence of the bacteriophage K genome is represented on the X-axes and the genomes of vB_Sau_Clo6, vB_Sau_CG and vB_Sau_S24 are represented on Y-axis. Gaps are zones with no homology, which correspond to the LTR region. (TIF) [file pone.0181671.s003.tif]

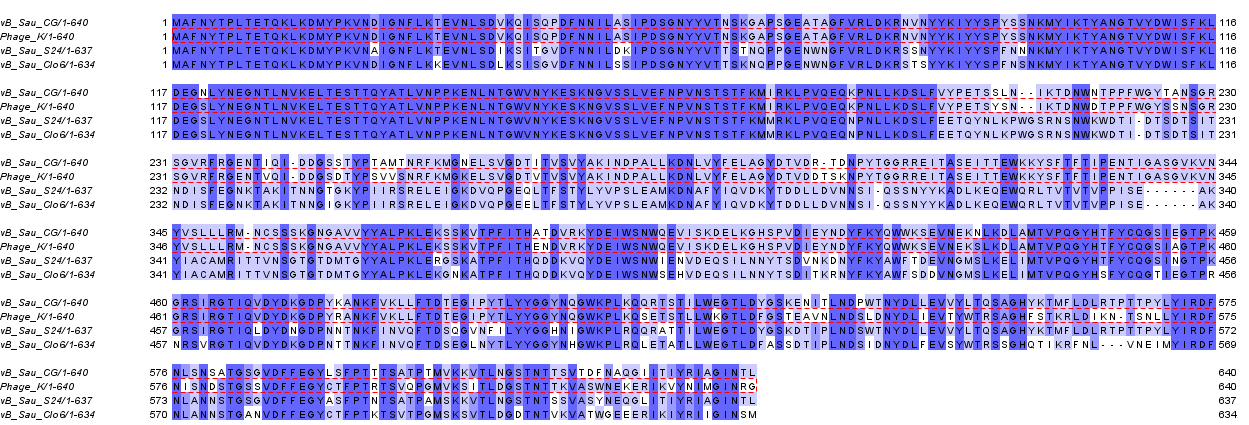

Supplement: S3 Fig — Alignment of the ORF41 of phages vB_Sau_S24 and vB_Sau_Clo6, ORF40 of vB_Sau_CG and gp146 of phage K was performed using Clustal Omega with default parameters. The results were visualized with Jalview 2.10.1 program; the color pattern shows percentage identity between proteins. (TIF) [file pone.0181671.s004.tif]
